# The characteristics of bone marrow-derived endothelial progenitor cells and their effect on glioma

**DOI:** 10.1186/1475-2867-12-32

**Published:** 2012-06-21

**Authors:** She-Hong Zhang, Ping Xiang, He-Yong Wang, You-Yi Lu, Yan-Li Luo, Hao Jiang

**Affiliations:** 1 Center for Laboratory Research, First Affiliated Hospital of Bengbu Medical College, Anhui 233004, China; 2 Center for Laboratory Research, First People’s Hospita, Shanghai Jiaotong University, Shanghai 200080, China; 3 Center for Laboratory Research, Pulmonary Hospital Affiliated Tongji University, Shanghai 200433, China; 4 Department of Radiation Oncology, First Affiliated Hospital of Bengbu Medical College, Anhui 233004, China

## Abstract

**Background:**

EPCs were isolated primarily in 1997 by Asahara et al. and recent studies indicated that bone-marrow-derived EPCs contributed little to the endothelium of tumor vessels. Tumors of the CNS system demonstrate various features of angiogenesis.

**Methods:**

EPCs derived from rat bone marrow were isolated and cultured in M199 medium without any induced factors. EPCs were studied using immunohistochemical staining, Flow cytometry and culture under three-dimensional condition to determine EPCs’ characteristics in vitro. We also established an animal model by injecting EPCs marked with Hoechst 33342 into the back of BALB/c nude mice and performed hematoxylin-eosin (HE) and immunofluorescent staining to study EPCs’ features in vivo. To research effect of EPCs on glioma, animals bearing tumors model with C6 glioma were established. About 27 day after injection, we performed immunohistochemical staining and Immunofluorescence staining.

**Results:**

Our results showed that EPCs derived from rat bone marrow appeared typical morphological characteristics and were positive of CD34, CD133, KDR and CD31 antigens at different time in vitro under the special M199 medium without any induced factors. The percentage of cells that expressed CD133 decreased gradually. In brief, the present study showed that EPCs derived from rat bone marrow differentiated into ECs in medium the without any induced factors and formed tubular structures in three-dimensional circumstances. Animal experiments suggested that EPCs differentiated into ECs and other else non-endothelial cells, and that EPCs contributed M199 of glioma.

**Discussion:**

These findings provides some novel results about biological characteristics of EPCs in vivo and ex vivo, and an update on the effect of EPCs on glioma and which would be helpful for the overall understanding of EPCs and make EPCs to be implied on the clinical therapy.

## Background

Since endothelial progenitor cells (EPCs) from peripheral blood (PB) were initially discovered and identified using the magnetic cells sorting method in 1997 by Asahara and his group [1], several researchers have found that EPCs existed in bone marrow, cord blood, and embryo-tissues, and that minimal number of EPCs also existed in heart, big blood vessel, skeletal muscle and fat tissue. So far, phenotypes of EPCs have remained to be determined, and exact differentiation has been still an ongoing debate topic. But it is widely accepted that early EPCs can be defined as CD133^+^/CD34^+^/KDR^+^ cells, whereas late EPCs are positive for CD34 and VEGFR2, lose CD133 and begin to express CD31, membrane molecules typical to mature ECs [1-3].

It was recently reported that EPCs derived from Embryonic Stem cells (ESCs) may be developed to use as a rapidly-expandable alternative for angiogenic transplantation therapy [4]. But EPCs derived from ESCs have not been studied extensively. We infer that the obstacle may be ethic problem. So we would rather select EPCs derived from rat marrow as our subject in the following study. Bone marrow-derived EPC played critical role in postnatal vasculogenesis through pivotal bioactivities, mobilization, homing, migration, differentiation, and proliferation in angiovasculogenic tissues [5]_._

Researchers generally accept that tumors are endowed with angiogenic-inducing capability, and critically dependent on blood vessels for their nutrient and oxygen delivery [6], allowing these cells to grow, invade tissue nearby, spread to the other parts of the body, and form new colonies of cancer cells [7,8], which idea came to light about fifty years ago when Judah Folkman and his group demonstrated that neovascularization is a necessary condition for malignant growth of solid tumors [9]. However, recent studies indicated that bone-marrow-derived cells contributed little to the endothelium of tumor vessels [10].

From the above review, the information about EPCs and tumors still remains a lot of arguments. We also observed that almost all of the previous studies were performed under the special induced circumstances. Instead, we have not seen any study of the characteristics of EPCs under the native condition. In the present study, we established a EPCs transplantation mice model and a mice model bearing glioma to further investigate BM-derived EPCs bioactivity and their effect on the growth of glioma in native circumstance without any induced factors.

## Materials and methods

All procedures were conducted in accordance with Chinese laws governing animal care, and were approved by the Institutional Animal Use and Care Committee of China.

### Isolation and culture of EPCs

The Ficoll density gradient centrifugation, a previously published method [11,12], was used to isolate mononuclear cells(MNCs) from rat bone marrow. Isolated mononuclear cells were cultured in complete medium 199 without any growth factors (M199, Gibco. American) special for EPCs in an atmosphere of humidified 5% CO_2_ at 37°C. Four-eight hours later, non-attached cells were reseeded and cultured. To observe EPCs’ morphology, we took photos for the cells during the period of culture.

### Cell proliferation assay

Cell proliferation was analyzed by 3-(4,5-dimethylthiazol-2-yl)-2,5-diphenyltetrazolium bromide (MTT, Gibco., American) assay as previously described [2]. In brief, 200ul medium per well containing 2 × 10^3^ cells were seeded in 96-well microtiter plates. Twenty-four hours later, cells were subjected to MTT assay, EPCs were supplemented with 20 ul MTT (5 mg/ml) and incubated for another 4 h. The supernatant was discarded and the EPCs preparations were shacked in 200ul dimethyl sulfoxide (DMSO, Gibco., American) for 10 min, followed by measurement of optical density value at 570 nm. Results from three independent experiments in triplicates were presented. The OD values were normalized to the blank control cells. We performed the above assay every other day until cells were grown to more than 95% confluency in the plates.

### Immunofluorescence

To determine the expression of CD34, CD133, KDR and CD31, the primary and 2nd passage EPCs were reseeded on glass coverslips in 6-well plate and were harvested several days later and fixed in 4% paraformaldehyde for 30 min. After washing three times with 1x PBS containing 1% Triton X-100 for 5 min, slides were then blocked with normal goat serum for 1 h at 4°C and then incubated with primary antibodies(rabbit anti-rat CD34, CD31, KDR and CD133 antibodies, Santa Cruz Co., American) (1:200) in a dark and moist chamber overnight at 4°C. After washing with 1x PBS for 3× 5 min, slides were incubated with relevant secondary antibodies (PE and FITC-conjugated goat anti-rabbit IgG, Santa Cruz Co., American) (1:500) for 1 h at room temperature. Slides were then washed three times with 1x PBS for 5 min and air dried, and covered onto glass slides with the mounting medium. The stained cells were observed under laser confocal scanning microscopy (Nikon Co., Japan) at once.

### Flow cytometry

To assess the dynamic change of surface antigen CD133 of cells, we performed fluorescence-activated cell sorter (FACS) analysis. Freshly-isolated MNCs were cultured for 10 days, 15 days, 20 days, 21 days and then harvested. The collected cells were blocked with normal goat serum for 1 h at 4°C and then incubated with goat anti-rat CD133 antibody (1:200) 4 h at 4°C. After washing with 1000rmp for 5 min, cells were incubated with FITC-conjugated goat anti-rabbit IgG (1:500) for 2 h at 4°C. After washing with 1000rmp for 5 min, cells were resuspended with 300ul PBS each tube and were examined quantitatively using Flow cytometry and CellQuest software (Becton DickinAson, Heidelberg, Germany) at once. The CD133-positive cells at different time were calculated as percentage of all MNCs. Each analysis included 100,000 events.

### Three-dimensional cell culture in vitro

A specific culture medium containing rat tail collagen for EPCs was used to make three-dimensional medium. 2 × 10^5^ EPCs were seeded in a 24-well plate in the middle of the two layers three-dimensional medium and cultured in an atmosphere of humidified 5% CO_2_ at 37°C. After 3 days of incubation, image of tubular structures were taken using invert light microscope with high power lens and the total number of tube formation at 400x visual fields for each well were calculated. Three separate experiments were performed.

### EPCs transplantation animal model

To study the characteristics of EPCs in vivo, the collected EPCs marked with Hoechst 33342 were utilized to establish a EPCs transplantation animal model. Briefly, 1 × 10^7^ EPCs were injected subcutaneously into both sides of the spine of 6-week-old SCID mice (n = 6). At the same time, PBS solution was also subcutaneously injected into both sides of the spine of 6-week-old SCID mice as a negative control (n = 6). We daily measured the volume of the subcutaneous tumor-like outgrowth formed by EPCs by a vernier caliper and calculated by the formula: Volume = L(Length) × w^2^(Width) × 0.52, which was previously published [13]. According to the above data, we drew a growth curve of the subcutaneous bulge.

After transplantation fifty days, the animals were euthanized and tumor-like outgrowth were removed and frozen in liquid nitrogen. Cryosections and paraffin section were prepared respectively for immunofluorescence staining with CD34, CD31, KDR antibodies and HE (hematoxylin-eosin) staining.

### Effect of EPCs on glioma

We utilized a rat C6 glioma transplantation model to study the effect of EPCs on glioma in vivo. In brief, Mixed cells suspensions containing 2 × 10^6^ rat C6 cells and 2 × 10^6^ EPCs/100 ul were injected subcutaneously into one flank of 6-week-old SCID mice (n = 10),called A group. At the same time, 2 × 10^6^ rat C6 cells were also subcutaneously injected into another flank of 6-week-old SCID mice as a negative control, called B group. Method of measuring the size of tumors was the same as described above.

After transplantation 27 days, the animals were euthanized and tumors were removed and frozen in liquid nitrogen and fixed in 10% formalin respectively. Cryosections and paraffin sections of 10 um were prepared respectively for immunofluorescence (ICH) staining with CD34, CD31, KDR antibodies and Immunohistochemistry staining.

Immunohistochemistry staining was used to detect if CD31 antigen expressed and how much expressed in the tumors exnografts. Immunohistochemistry processes was accorded to the instruction of the reagent kit. The goat-anti-rat CD31 antibody (1:200) was applied as primary antibody. Peroxidase -conjugated goat-anti-rabbit IgG (1:500) as secondary antibody.

Stained tumor sections were scanned at low power, and the areas of greatest CD31-positive density were chosen for quantification of vessel density. In addition, to evaluate the quantitative change in tumor vasculature, micro-vascular density (MVD) was estimated on CD31 immunostained sections. The number of micro-vessels was counted under microscope in eight fields and the mean MVD was calculated to the average number of micro-vessels.

## Results

### Morphological features of EPCs

**Figure 1:**
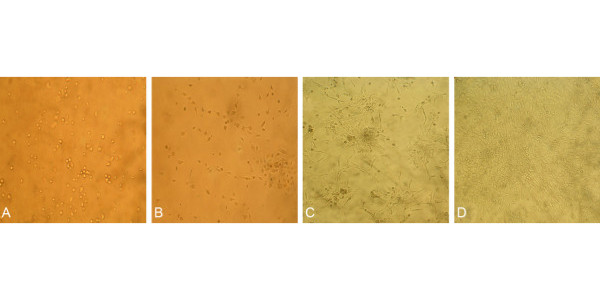
**(10×) A. the appearance of adherented EPCs within 24h; B. CFUs and Linear structure formed by EPCs at the 4th day; C. CFUs were connected with each other at the 7th days; D. EPCs grew quickly and covered the bottom of the flask with cobblestone-like appearance at the 11th days.**

After culture for 24 h, a few of cells attached to the culture flask, cells were characterized by conglobate cell body and clear cytoplasm (Figure 1A). During 2–6 days of culture, cells became spindle-shape. Some of cells began to connect with each other and exhibited linear cord-like structures (Figure 1B). After 6 days of culture, the adjacent cells continued to connect and formed colonies (Figure 1C). Approximately 11 days after MNCs were cultivated, cells colonies gradually reached 100% confluence and appeared typical cobblestone-like monolayer (Figure 1D).

### Proliferation of EPCs

**Figure 2:**
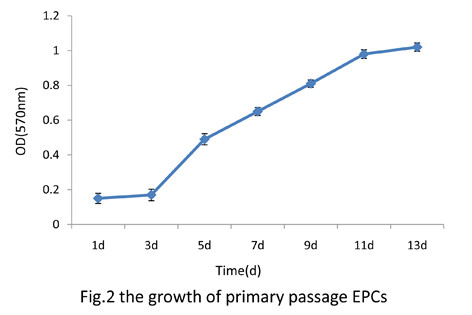
The growth curve of EPCs.

The primary EPCs showed the following growth feature: during 1 to 3 days of culture, cells grew slowly. From 4 to 11 days of culture, cells grew quickly. After that, cells grew slowly again (Figure 2).

### Immunofluorescence staining of EPCs

**Figure 3:**
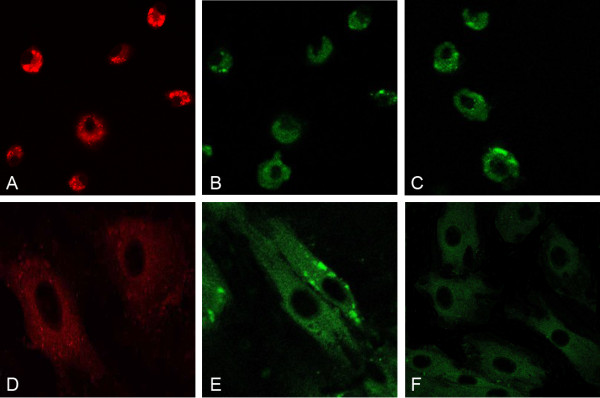
A–C(10×) At the 2nd day, cells expressed antigen KDR (Red), CD133(Green),CD34(Green) respectively; D–F(40×) At the 21st day, cells expressed antigen KDR (Red), CD31(Green),CD34(Green) respectively.

The primary passage EPCs were collected and reseeded on the glass coverslips in 6-well plate. After 2 days of culture, cells were used to identify the surface antigen (CD34, CD133 and KDR). Data showed that cells were positive with the above three antigens (Figure 3A-C). The 2nd passage EPCs were collected and reseeded on the glass coverslips in 6-well plate. After 2 days of culture, cells were used to identify the surface antigen of cells (CD34, CD31 and KDR). Data showed that cells were positive with the above three antigens (Figure 3D-F).

### The dynamic change of surface antigen CD133 of cells

**Figure 4:**
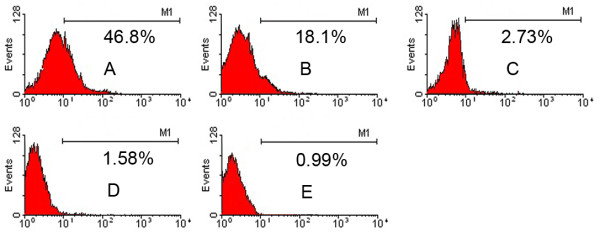
**A–D Marrow-derived EPCs were detected by Flow cytometry and expressed antigen CD133 at the 10th, 15th, 20th, 21st day.** 4**E**. It was the negative control.

At the day 10,15,20,21, CD133-positive rate of MNCs were 46.8%,18.1%,2.73%,1.58% respectively (Figure 4A-D). However, the control was only 0.99% (Figure 4E). With the time flied away, CD133-positive rate became gradually lower till lost. After culture for 20 days, the percentage of cells that expressed CD133 decreased dramatically compared with that of the 15 days (P<0.05).

### Tubular structures formed by EPCs in vitro

**Figure 5:**
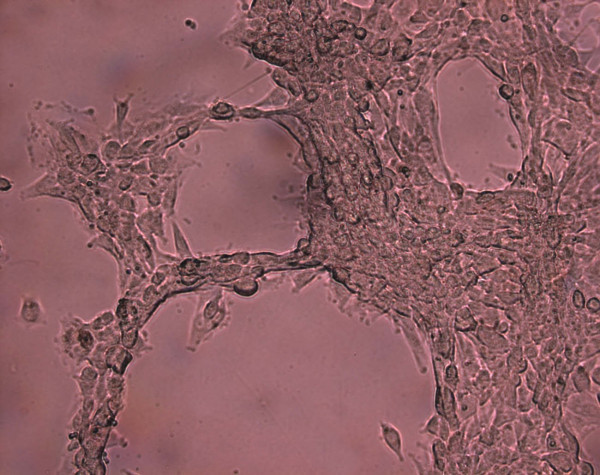
(40×) Three-dimensional cells culture, tube-like structures were observed.

On day 5 of Three-dimensional cell culture, tube-like structures were observed by invert light microscope with high power lens (Figure 5). There were 3 tube-like structures on each visual field (40×).

### Subcutaneous formation of EPCs in vivo

**Figure 6:**
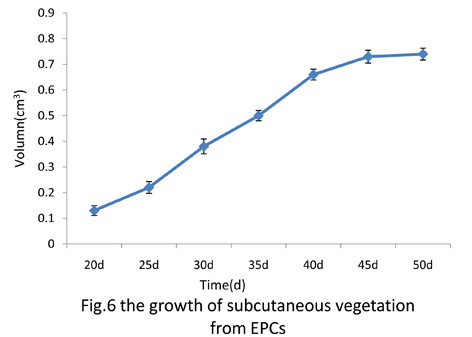
The growth of subcutaneous vegetation from EPCs.

After 20 days transplantation, subcutaneous outgrowth with the volume of 0.13 cm^3^ formed by EPCs was observed, and then grew bigger and bigger until the animals were euthanized and tumor-like formations were removed at 50 day. According to the volume of the subcutaneous outgrowth from EPCs, we drew a growth curve of it (Figure 6), data showed that EPCs xenografts seemed to be aging.

### Differentiation of EPCs in vivo

**Figure 7:**
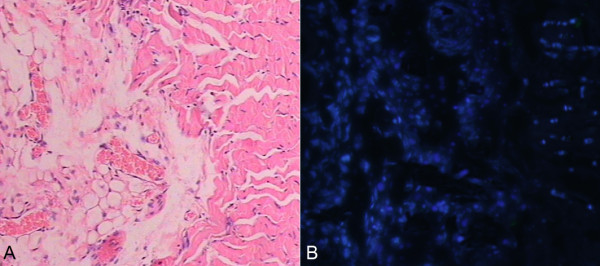
**A–B HE staining of subcutaneous vegetation from EPCs, at the same field cells marked with Hoechst 33324 were observed.** (10×) **A**. HE staining of the subcutaneous processes; **B**. EPCs marked with Hoechst 33342.

Paraffin sections were performed HE (hematoxylin-eosin) staining, data showed that there were three kinds of structures of samples, they were vessel-like endothelial network formation, muscle-like tissue and fat-like cells respectively (Figure 7A). Meanwhile, we observed cells marked with Hoechst 33324 (Figure 7B).

### Immunofluorescence staining of exnograft derived from donors EPCs

**Figure 8:**
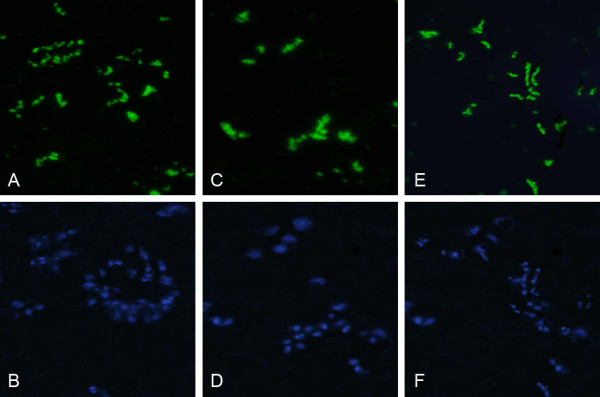
**A/B(20×) A. cells expressed antigen CD34(green); B. cells labled with Hoechst 33342(blue).C**. cells expressed antigen Flk1(green); **D**. cells labled with Hoechst 33342(blue). **E**. cells expressed antigen CD31(green); **F**. cells labled with Hoechst 33342(blue).

The immunofluorescence assay revealed, firstly, both CD 34 and Hoechst 33342 positive cells appeared in the same visual (Figure 8A-B). Secondly, EPCs, which expressed both CD 31 and Hoechst 33342 positive cells located in the tumor (Figure 8C-D). Finally, both Flk1 and Hoechst 33342 positive cells were observed in the same visual too (Figure 8E-F). It also proved that EPCs expressed blue fluorescence were donor-derived endothelial progenitor cells.

The data showed that EPCs derived from donors could differentiate into endothelial cells, which expressed CD34, KDR and CD31 antigens, in the SCID-mice. But we also observed that cells marked with Hoechst 33342 did not express the above three antigens, which suggested that some of EPCs derived from donors must have been able to differentiate into other else non- endothelial cells.

### Growth of the transplantation tumor

**Figure 9:**
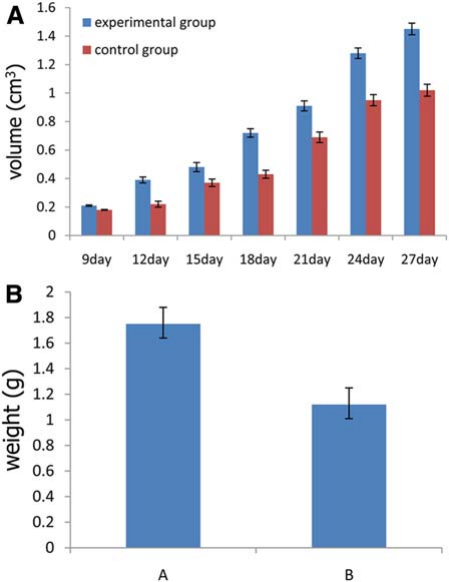
**A the volume of tumors of A group and B group at the 27th day(P<0.05). B** the weight of tumors of A group and B group at the 27th day(P<0.05).

About 9 days after C6 glioma cells with or without EPCs were injected into animals, subcutaneous tumors were observed. The mean volume of the experimental group tumor was (0.21±0.005)cm^3^, the control group was (0.18±0.003)cm^3^. After that, the volume of the tumors were measured every 3 days. After transplantation 27 days, the animals bearing tumor were euthanized and tumors were removed. As summarized in Figure 9A, the size of tumors of A group were significant bigger compared to the control, B group(P<0.05), and at the same time, the quality of A group was (1.75±0.13)g, while the quality of B group was (1.12±0.11)g (Figure 9B)(P<0.05).

### Immunohistochemistry staining of glioma exnografts

**Figure 10:**
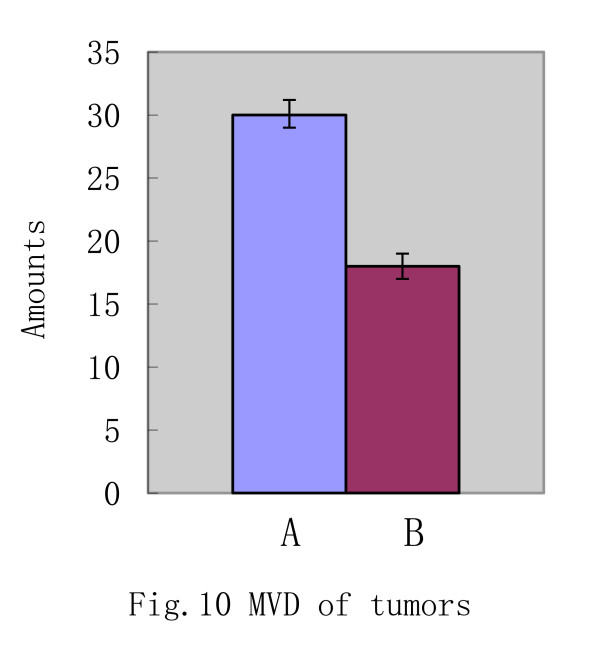
The microvascular density of tumor of the test group and the control group at the 27th day(P<0.05).

**Figure 11:**
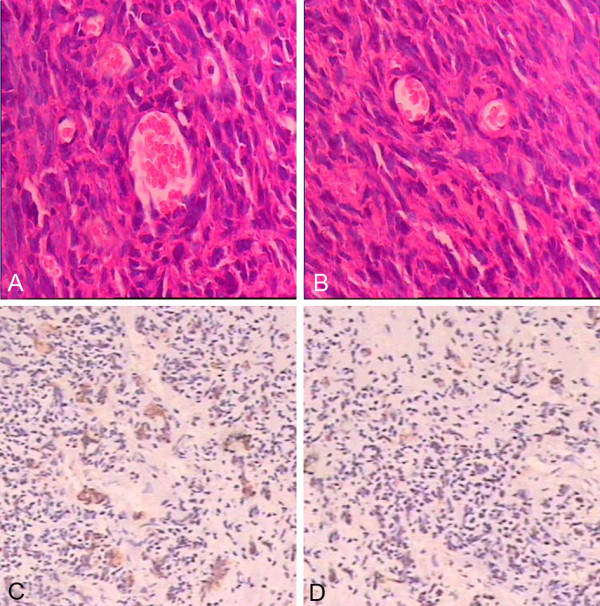
**A–B (20×) HE staining of A and B group tumors.C**–**D** (10×) antigen CD31 immunohistochemistry staining in the A and B group.

About 27 days after cells were injected subcutaneously into mice, huge tumors were harvested from two groups mice. To evaluate the mean microvascular density (MVD) of the tumors, we performed immunohistochemistry staining with CD31 antibody and quantified the rate of CD31 positivity. Data revealed that tumors exhibited brown cytoplasmic staining in both controls and experimental groups, which suggested that tissues of the tumors expressed positively CD 31 antigen. We selected five different microscopic fields at random and counted the number of CD31-positive cells per field. (30 ± 1.2) cells were CD31-positive in A group, whereas (18 ± 1.0) cells were positive for CD31 in B group. According to the brown staining expression, we evaluated the mean microvascular density (MVD), the above results indicated that EPCs significantly increased the extent of MVD in the tumors (p<0.05) (Figure 10). In addition, immunohistochemical data from the xenograft studies were presented (Figure 11A-B).

### Immunofluorescence assay of glioma exnografts

**Figure 12:**
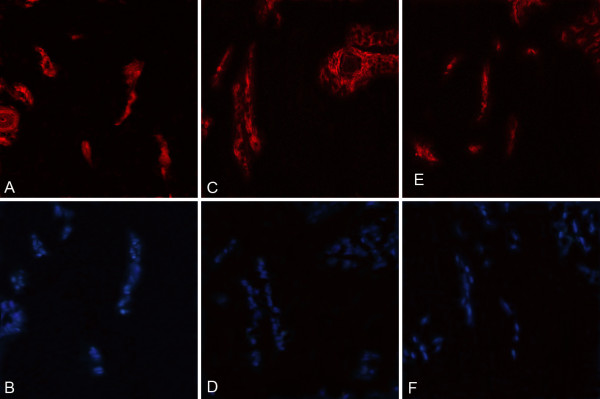
**A/B(20×) cells of the test group tumor tissue expressed antigen CD31(red)and Hoechst 33342(blue). C**/**D**(20×) cells of the test group tumor tissue expressed antigen Flk1(red) and Hoechst 33342(blue). **E**/**F**(20×) cells of the test group tumor tissue expressed antigen CD133(red) and Hoechst 33342(blue).

Immunofluorescence staining showed that cells, expressed simultaneously CD34,CD133,KDR and Hoechst 33342, appeared in the tumors (Figure 12A-F), which proved that EPCs derived from donors incorporated into the tumors and contributed to the growth of glioma.

## Discussion

The present study demonstrated that MNCs could be separated by a method of Ficoll density gradient centrifugation from rat bone marrow, consistent with previous reports [10,11]. EPCs were isolated primarily in 1997 by Asahara et al. [1], and since then has been extensively studied not only in biologic characteristics but functional implication. EPCs have been shown to express the endothelial cell surface antigens CD34, KDR and stem cell surface antigens CD133, and uptake AcLDL, so we can isolate or identify by these markers [11-15]. EPCs could be counted as CD34+ CD45^low^ CD133+ VEGFR2+ cells by flow cytometry [16]. The recent study reported that besides of the above markers, EPCs expressed also VDR, and cells co-expressed CD34(+)/CD133(+/−)/KDR(+) and VDR seem to be influenced by uremia-related factors, including anemia, inflammation, diabetes, 25(OH)D serum levels and calcitriol therapy [17]. In the current study, EPCs derived from rat bone marrow expressed CD34, CD133 and Flk1/KDR/VEGFR2, which were proved by Immunohistochemical staining.

Flow cytometry analysis also revealed that during the procedure of culture, another stem cell marker, CD133, was in the dynamic changing state, which was only expressed at the early period of MNCs culture but was gradually reduced thereafter, indicating that CD133 seems to be a marker of early EPCs in bone marrow. In contrast, several recent studies suggested that CD31 was little expressed initially (i.e., in freshly isolated circulating MNCs) but gradually appeared after cultured for several passages. These results suggested that CD133 seems to be a marker of early EPCs, whereas CD31 will be used as a marker for differentiated EPCs.

The typical morphology features of EPCs, adherent colonies with a cobblestone profile, also appeared in our study, which is consistent with that described previously [1,18]. Furthermore, another independent experiment, three dimensional cultured in a rat tail collagen stereo model in vitro, proved that functional EPCs could be expanded from the primary culture of MNCs obtained from rat bone marrow under M199 medium without any induced factors, and that culture-expanded EPCs could developed into tube-like network formation in vitro, which is an interesting finding and not reported up to now.

Another interesting finding of the present study is as follows. We employed an immunodeficient nude mouse as a transplanted subject and EPCs-derived from bone marrow were injected subcutaneously into both sides of spinal column of an immunocompromised mouse. After fifty days, tumor-like outgrowth from transplanted EPCs are harvested and then investigated. The results indicated that EPCs-derived from bone marrow could differentiate into endothelial cells and non- endothelial cells, such as muscle-like cells and fatty-like cells without any induced factors. However, we cannot answer what is their precise identification, which is one of the major limitations of the current study.

Previous studies indicated that endothelial progenitor cells (EPCs) in bone marrow and peripheral blood contributed to tissue repair in various pathological conditions via the formation of new blood vessels [19]. Nevertheless, it has been documented that EPCs could specially home and incorporate into sites of physiological vessel formation in vivo and incorporate into the vasculature of tumors, ischemic skeletal and cardiac muscle, and ulcers [20-22]. It is well known that the pivotal process of tumor growth and metastasis is angiogenesis. In clinical, more and more anti-angiogenesis drugs have been developed to control this process [23,24]. Angiogenesis is a complex process and its mechanisms still remain unclear. Malignant glioma tends to be highly vascularized, particularly glioblastoma, which are associated with florid microvascular proliferation [25]. We also demonstrated that transplantation of both culture-expanded EPCs from MNCs and C6 glioma augmented neovascularization in experimental animals-bearing tumor, which was similar with other reports [26,27], though the culture condition of EPCs is different between our study and other’. We also found that previous studies all employed rat tail collagen fibronectin matrix as solid braces injected subcutaneously into the subject animals, but it was reported that Matrigel could induce cord formation from several non-endothelial cells including fibroblasts [28], baby murine kidney cells [29], aortic smooth muscle cells, murine leydig cells [30], and CD14^+^ monocytes seeded in Matrigel [31].

In summary, the current paper provides some novel findings about biological characteristics of EPCs in vivo and ex vivo, and an update on the effect of EPCs on glioma. We believe that these data would be helpful for the overall understanding of EPCs and make EPCs to be implied on the clinical therapy.

## Competing interests

The authors declare that they have no competing interests.

## Authors’ contribution

ZSH participated in the design of the study, carried out all the studies and drafted the manuscript. 2) XP conceived of the study, provided all the funds, participated in its design and coordination and helped to draft and revise the manuscript. 3) WHY, LYY, LYL and JH participated in the animal study and involved in drafting the manuscript. All authors read and approved the final manuscript.
